# Cardiac‐specific overexpression of caveolin‐3 preserves t‐tubular *I*_Ca_ during heart failure in mice

**DOI:** 10.1113/EP087304

**Published:** 2019-03-14

**Authors:** Cherrie H. T. Kong, Simon M. Bryant, Judy J. Watson, David M. Roth, Hemal H. Patel, Mark B. Cannell, Andrew F. James, Clive H. Orchard

**Affiliations:** ^1^ School of Physiology Pharmacology & Neuroscience Biomedical Sciences Building University of Bristol Bristol BS8 1TD UK; ^2^ VA San Diego Healthcare System and Department of Anesthesiology University of California San Diego, La Jolla CA USA

**Keywords:** caveolin‐3, excitation–contraction coupling, overexpression, t‐tubules, TAC

## Abstract

**New Findings:**

- **What is the central question of this study?**
  What is the cellular basis of the protection conferred on the heart by overexpression of caveolin‐3 (Cav‐3 OE) against many of the features of heart failure normally observed *in vivo*?
- **What is the main finding and its importance?**
  Cav‐3 overexpression has little effect in normal ventricular myocytes but reduces cellular hypertrophy and preserves t‐tubular *I*_Ca_, but not local t‐tubular Ca^2+^ release, in heart failure induced by pressure overload in mice. Thus Cav‐3 overexpression provides specific but limited protection following induction of heart failure, although other factors disrupt Ca^2+^ release.

**Abstract:**

Caveolin‐3 (Cav‐3) is an 18 kDa protein that has been implicated in t‐tubule formation and function in cardiac ventricular myocytes. During cardiac hypertrophy and failure, Cav‐3 expression decreases, t‐tubule structure is disrupted and excitation–contraction coupling (ECC) is impaired. Previous work has suggested that Cav‐3 overexpression (OE) is cardio‐protective, but the effect of Cav‐3 OE on these cellular changes is unknown. We therefore investigated whether Cav‐3 OE in mice is protective against the cellular effects of pressure overload induced by 8 weeks’ transverse aortic constriction (TAC). Cav‐3 OE mice developed cardiac dilatation, decreased stroke volume and ejection fraction, and hypertrophy and pulmonary congestion in response to TAC. These changes were accompanied by cellular hypertrophy, a decrease in t‐tubule regularity and density, and impaired local Ca^2+^ release at the t‐tubules. However, the extent of cardiac and cellular hypertrophy was reduced in Cav‐3 OE compared to WT mice, and t‐tubular Ca^2+^ current (*I*_Ca_) density was maintained. These data suggest that Cav‐3 OE helps prevent hypertrophy and loss of t‐tubular *I*_Ca_ following TAC, but that other factors disrupt local Ca^2+^ release.

## 1 INTRODUCTION

Excitation–contraction coupling (ECC) in cardiac myocytes is initiated by the action potential, which activates sarcolemmal L‐type Ca^2+^ channels (LTCCs). The consequent Ca^2+^ influx (*I*_Ca_) triggers Ca^2+^ release from adjacent sarcoplasmic reticulum (SR) via Ca^2+^ release channels (ryanodine receptors; RyRs). This Ca^2+^‐induced Ca^2+^ release (Fabiato, 1985) produces local increases of cytosolic [Ca^2+^] (‘Ca^2+^ sparks’; Cheng, Lederer, & Cannell, 1993) that summate to form the cytosolic Ca^2+^ transient, leading to contraction. In ventricular myocytes, *I*_Ca_, and thus RyR activation, occurs predominantly at specialized invaginations of the sarcolemma called t‐tubules (Cannell, Cheng, & Lederer, 1994; Kawai, Hussain, & Orchard, 1999; Lindner, 1957). This arrangement achieves near‐synchronous Ca^2+^ release (Cheng, Cannell, & Lederer, 1994), and thus contraction, throughout the cell. Relaxation occurs as cytosolic [Ca^2+^] decreases, mainly due to re‐uptake into the SR, but also by removal from the cell via Na^+^–Ca^2+^ exchange (Negretti, O'Neill, & Eisner, 1993).

Caveolin‐3 (Cav‐3) is a cholesterol‐binding protein that has been implicated in the genesis of t‐tubules (Parton, Way, Zorzi, & Stang, 1997) and in the localization of LTCC regulatory proteins and *I*_Ca_ to the t‐tubules (Balijepalli, Foell, Hall, Hell, & Kamp, 2006; Bryant et al., 2014). Cav‐3 knock‐out (KO) leads to cellular hypertrophy, t‐tubule disorganization and decreased t‐tubular *I*_Ca_ density (Bryant et al., 2018b). Disruption of Cav‐3 signalling with C3SD peptide (Couet, Li, Okamoto, Ikezu, & Lisanti, 1997; Feron et al., 1998) also decreases t‐tubular *I*_Ca_ (Bryant et al., 2014), which impairs local SR Ca^2+^ release (Bryant et al., 2014; Bryant et al., 2018a). Interestingly, cardiac hypertrophy and heart failure (HF) are associated with decreased Cav‐3 expression (Bryant et al., 2018a), and myocytes from such hearts also show hypertrophy, t‐tubular disruption, decreased t‐tubular *I*_Ca_ density and impaired SR Ca^2+^ release (Bryant et al., 2015; Bryant et al., 2018a), suggesting that reduced Cav‐3 expression may play a role in the phenotypic changes observed in these conditions. In support of this idea, Cav‐3 KO results in a progressive cardiomyopathy characterized by ventricular hypertrophy and dilatation and reduced fractional shortening (Woodman et al., 2002), while a loss‐of‐function mutation in Cav‐3, T63S, has been associated with inherited hypertrophic cardiomyopathy (Hayashi et al., 2004). In addition, overexpression (OE) of Cav‐3 reduces the functional and phenotypic changes caused by pressure overload induced by transverse aortic constriction (TAC; Horikawa et al., 2011; Markandeya et al., 2015), which normally results in cardiac hypertrophy and failure. However, the cellular changes underlying this cardioprotection remain unclear. The present study was undertaken, therefore, to investigate how Cav‐3 OE alters the response of ventricular myocyte structure and ECC to TAC in mice.

## 2 METHODS

### 2.1 Ethical approval

All animal procedures were approved by the Animal Welfare and Ethics Review Board of the University of Bristol (14/6/2016) and conducted in accordance with UK legislation (Animals (Scientific Procedures) Act 1986 Amendment Regulations 2012 incorporating European Directive 2010/63/EU); the study also complies with the ethical principles under which *Experimental Physiology* operates.

### 2.2 Animals and surgical procedures

Adult (12 week) male homozygous cardiac‐specific Cav‐3 OE mice, produced as described previously (Tsutsumi et al., 2008), were used. Data from these mice, bred at the University of Bristol, are compared with data obtained from wild‐type (WT) littermates that had undergone either sham operation or TAC that resulted in HF. The WT data have been published previously (Bryant et al., 2018a) so that only mean data, rather than original records, are shown for the WT group, for comparison with the OE data. However, the surgical and experimental procedures were the same, and performed contemporaneously, for the WT and Cav‐3 OE groups, and data were obtained using the same techniques and protocols in each group, as described below. Surgery was performed at 12 weeks of age and myocyte isolations at 20 weeks of age. Mice were kept in a temperature‐controlled, enriched environment with *ad libitum* access to food and water.

Eight weeks of TAC was used to produce pressure overload, since this has previously been shown to result in cardiac hypertrophy and failure (Bryant et al., 2018a; Rockman et al., 1991; Tachibana, Naga Prasad, Lefkowitz, Koch, & Rockman, 2005). Briefly, animals were anaesthetized with ketamine (75 mg kg^−1^ i.p., Zoetis UK Limited, London, UK) and medetomidine (1 mg kg^−1^ i.p., Orion Corp., FI‐02200 Espoo, Finland) and given buprenorphine (0.05 mg kg^−1^ s.c., Reckitt Benckiser Health Care (UK) Ltd, Hull, UK) for pain relief; the surgical plane of anaesthesia was monitored using the limb withdrawal reflex. The aortic arch was exposed via a medial sternal thoracotomy and a silk ligature (6‐0) placed between the innominate and left carotid arteries and tied round a 27G needle (0.4 mm OD). Sham animals underwent the same operation but without placement of the banding suture. Animals were maintained post‐operatively for 8 weeks before use.

### 2.3 Echocardiography

*In vivo* cardiac structure and function were monitored using echocardiography. Animals were anaesthetized (isoflurane 1–3%, Merial Animal Health Ltd, Harlow, UK), heart rate was monitored, and measurements of contractile performance made from M‐mode images acquired from the parasternal short axis view using a Vevo 3100 (Fujifilm VisualSonics Inc., Toronto, Ontario, Canada) and MX550D transducer.

### 2.4 Myocyte isolation and detubulation

Animals were killed by cervical dislocation and ventricular myocytes isolated using standard enzymatic digestion via Langendorff perfusion as described previously (Bryant et al., 2014) and used on the day of isolation. Detubulation (DT), the physical and functional uncoupling of the t‐tubules from the surface membrane, was achieved using formamide‐induced osmotic shock as described previously (Brette & Orchard, 2003; Brette, Komukai, & Orchard, 2002; Kawai et al., 1999); comparison of membrane capacitance and currents in intact and detubulated myocytes enables the distribution of membrane currents and current density between the t‐tubule and surface membranes to be determined.

### 2.5 Imaging and analysis of t‐tubule structure

Cell width and length were measured from brightfield images of isolated myocytes used for electrophysiology. Cell volume was calculated from these measurements as described previously (Boyett, Frampton, & Kirby, 1991).

Surface and t‐tubular cell membranes were labelled by incubating cells with 5 μmol l^−1^ di‐8‐ANEPPS for 10 min. Image volumes were obtained using an LSM 880 confocal microscope (Zeiss, Carl Zeiss AG, Oberkochen, Germany) in Airyscan ‘super‐resolution’ mode, with a 1.2 NA, ×40 water immersion objective, sampled at 40 nm in‐plane and 180 nm along the optical axis. Airyscan uses a 32‐channel photomultiplier tube detector that collects a pinhole‐plane image at every scan position, thus improving spatial resolution. In super‐resolution mode, linear deconvolution provides further improvement to achieve spatial resolution that is 1.7× that of a conventional confocal microscope. The regularity of t‐tubule staining was quantified by applying a two‐dimensional (2D) fast Fourier transform (FFT) to an offset‐subtracted square region of the cell interior, and the power of the first harmonic normalized to that of the average image intensity (*P*_1_/*P*_0_). T‐tubule density was calculated from an intracellular volume marked by hand. The 3D skeleton of the t‐tubules was obtained by processing the volumetric data with a tubule‐enhancing 3D filter, segmenting using an Otsu threshold in MATLAB R2015a (The Mathworks Inc., Natick, MA, USA), and converting to a skeleton using Skeletonize (2D/3D) in ImageJ (v1.50, NIH, Bethesda, MD, USA). The skeleton was used to calculate t‐tubule density (skeleton length divided by the marked intracellular volume, μm μm^−3^) and local Eigenvectors for t‐tubule angles. Tubule orientation is expressed relative to the transverse plane, so that 0° corresponds to a transverse tubule, while 90° corresponds to a tubule that extends along the cell (i.e. an ‘axial’ tubule).

### 2.6 Western blotting

Following myocyte isolation, aliquots were pelleted by centrifugation, the supernatant removed and the cell pellet snap frozen in liquid nitrogen and stored at −80°C. Once all samples had been acquired, the pellets were processed simultaneously by thawing directly into lysis buffer containing 50 mm Tris–HCl pH 7.4, 150 mm NaCl, 1 mm EDTA, 1% (v/v) Triton X‐100, 1% (w/v) sodium deoxycholate, 0.1% (v/v) SDS, 1 mm phenylmethylsulfonyl fluoride, complete protease inhibitors, 8 μg ml^−1^ calpain inhibitor I, 8 μg ml^−1^ calpain inhibitor II, 50 mm sodium fluoride, 1 mm sodium orthovanadate, and 16 mm sodium pyrophosphate, homogenized by pipetting and incubated on ice for 15 min. After centrifugation at 13,000 *g* 4°C for 15 min the supernatants were collected, protein concentrations estimated using the Pierce BCA protein assay (Thermo Fisher Scientific, Waltham, MA, USA) and adjustments made to allow for equal protein loading on SDS‐PAGE. Ten‐microgram samples of the myocyte lysates were run on 4–15% gradient SDS‐PAGE gels and transferred onto Immobilon‐P membrane. Blots were probed with antibodies against Cav‐3 (BD Biosciences, San Jose, CA, USA, cat. no. 610420, dilution 1:5000), junctophilin‐2 (JPH‐2; Thermo Fisher Scientific, Waltham, MA, USA, cat. no. 40–5300, dilution 1:500) and glyceraldehyde 3‐phosphate dehydrogenase (GAPDH) (Sigma‐Aldrich, St Louis, MO, USA, cat. no. G9545, dilution 1:100,000). Protein bands were visualized and images captured using horseradish peroxidase‐conjugated secondary antibodies (Promega, Madison, WI, USA; cat. no. W4011, α‐rabbit HRP, dilution 1:10,000 and cat. no. W4021, α‐mouse HRP, dilution 1:10,000), chemiluminescence and a G:BOX Chemi XT4 imaging system (Syngene, Cambridge, UK). Gels were first probed with the antibody to Cav‐3 or JPH‐2, then stripped using a commercial stripping solution (Restore™ western blot stripping buffer, Thermo Fisher Scientific) and re‐probed with the loading control antibody to GAPDH, before being stripped and re‐probed for JPH‐2 or Cav‐3. The density of the bands was measured using ImageJ and normalized to GAPDH.

### 2.7 *I*_Ca_ recording

Myocytes were placed in a chamber mounted on a Nikon Diaphot inverted microscope. Membrane currents and cell capacitance were recorded using the whole‐cell patch‐clamp technique, using an Axopatch 200B, Digidata 1322A A/D converter and pClamp 10 (Molecular Devices, LLC, San Jose, CA, USA). Pipette resistance was typically 1.5–3 MΩ when filled with pipette solution (see below), and pipette capacitance and series resistance were compensated by ∼70% to optimize the measurement of membrane current. Currents were activated from a holding potential of −80 mV by step depolarization to −40 mV for 200 ms (to inactivate the sodium current) followed by steps to potentials between −50 and +80 mV for 500 ms, before repolarization to the holding potential, at a frequency of 0.2 Hz. Absolute *I*_Ca_ amplitude (pA) in intact myocytes was measured as the difference between peak inward current and current at the end of the depolarizing pulse; absolute *I*_Ca_ in the t‐tubular and surface membranes was calculated from measurements of *I*_Ca_ and membrane capacitance in intact and detubulated myocytes with correction for incomplete detubulation as described previously (Bryant et al., 2015). *I*_Ca_ was normalized to cell capacitance (pF; an index of membrane area) to calculate *I*_Ca_ density (pA pF^−1^). *I*_Ca_ density in the t‐tubule membrane was calculated from the loss of membrane current and capacitance following DT; *I*_Ca_ density in the surface membrane was calculated from currents measured in DT myocytes with correction for incomplete detubulation as described previously (Bryant et al., 2015). DT efficiency was not significantly different between cell types.

### 2.8 Measurement of SR Ca^2+^ release

Intracellular Ca^2+^ and membrane potential were recorded simultaneously along single t‐tubules as described previously (Bryant et al., 2015). Briefly, myocytes were loaded with the Ca^2+^ indicator Fluo‐4/AM (5 μmol l^−1^ for 25 mins; Thermo Fisher Scientific) and the voltage sensitive dye di‐4‐AN(F)EPPTEA (0.5–1 μg ml^−1^ for 15 min; kindly supplied by Dr Leslie Loew; Yan et al., 2012). Cells were imaged using a Zeiss LSM 880 (see above) with the confocal pinhole set to 1 Airy unit. Line‐scan images along a selected t‐tubule were recorded at wavelengths between 518 and 560 nm for Ca^2+^, and 590 and 700 nm for voltage, at a rate of 0.51 ms/line, with an excitation wavelength of 514 nm. A negative deflection in di‐4‐AN(F)EPPTEA fluorescence was used to determine the time of the AP upstroke, and the latency from the AP upstroke to the initial (>5 SD above average pre‐stimulus value) and maximum rate of rise of Ca^2+^ was determined at each point along the Fluo‐4 line‐scan image. The SD of latencies for each cell was used as a measure of the heterogeneity of release. Whole‐cell Ca^2+^ transients were obtained using line‐scans along the long axis of cells loaded with Fluo‐4/AM only. Cells were field‐stimulated at 0.2 Hz at 1.5 × threshold using parallel Pt electrodes.

### 2.9 Solutions

The standard superfusate for electrophysiology and imaging experiments contained (in mm): 133 NaCl, 5 KCl, 1 MgSO_4_, 1 CaCl_2_, 1 Na_2_HPO_4_, 10 d‐glucose, 10 HEPES, pH 7.4 (NaOH). During electrophysiological recordings, KCl was replaced with CsCl to inhibit K^+^ currents and the pipette solution contained (in mm): 110 CsCl, 20 TEACl, 0.5 MgCl_2_, 5 MgATP, 5 BAPTA, 10 HEPES, 0.4 GTP‐Tris, pH 7.2 (CsOH). For some experiments, cells were incubated in C3SD peptide (Pepceuticals Limited, Enderby, UK, 1 μm in 0.1 mm Ca^2+^) for 1 h at room temperature before use. C3SD was designed to mimic the ‘scaffolding domain’ of Cav‐3 and thus to interfere with Cav‐3 binding to its endogenous partners and, as used in the present study, has previously been shown to inhibit Cav‐3‐dependent signalling in cardiac myocytes (Bryant et al., 2018b). All experiments were performed at room temperature.

### 2.10 Data presentation

Data are expressed as mean ± SD (of *N* animals for *in vivo* data and of *n* cells from *N* animals (*n*/*N*) for cellular measurements). Data normality was assessed using the Shapiro–Wilk test and subsequent testing was performed using Student's *t* test or the Mann–Whitney *U* test, one‐way ANOVA, or the Kruskal–Wallis test, as appropriate. *I*_Ca_ density–voltage relationship curves were analysed using repeated measures (RM) ANOVA with voltage and intervention as factors. Single myocyte properties including those elicited by a step depolarization to a single voltage were analysed with two‐way ANOVA; *post hoc* tests used the Bonferroni correction. The errors in derived variables (specifically *I*_Ca_ density at the t‐tubule and surface membranes), and the subsequent statistical analysis (unpaired Student's *t* test), were calculated using propagation of errors from the source measurements (Bryant et al., 2015). The limit of statistical confidence was taken as *P *< 0.05, and is denoted by * between treatments (e.g. sham *vs*. TAC) for a given phenotype and by † between phenotypes (WT *vs*. Cav‐3 OE).

## 3 RESULTS

### 3.1 The effect of TAC on cardiac structure and function

**Figure 1:**
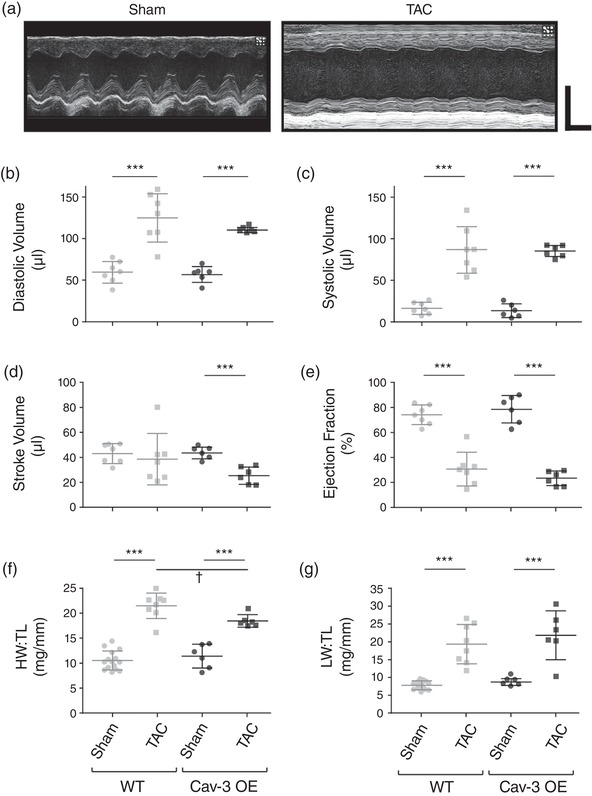
*In vivo* cardiac function and morphology. (a) Exemplar left ventricular M‐mode echocardiograms from Cav‐3 OE mice that had undergone either a sham (left panel) or TAC (right panel) operation. Horizontal scale bar: 100 ms; vertical scale bar: 4 mm. (b–e) Measurements of left ventricular function obtained by echocardiography from sham and TAC WT (left) and Cav‐3 OE (right) mice. (b) Diastolic volume (μl). (c) Systolic volume (μl). (d) Stroke volume (μl). (e) Ejection fraction (%). (f) Heart weight to tibia length ratio (HW:TL, mg/mm). (g) Lung weight to tibia length ratio (LW:TL, mg/mm). *N* = 6 sham and 6 TAC Cav‐3 OE mice, and 7 sham and 7 TAC WT mice. ***P < 0.001 between treatments for a given phenotype (WT or Cav‐3 OE); †*P *< 0.05 between phenotypes for a given treatment (sham or TAC). The WT data have been published previously (Bryant et al., 2018a)

Cardiac structure and function were assessed *in vivo* using echocardiography; exemplar records from Cav‐3 OE mice are shown in Figure 1a; WT data have been shown previously (see Methods). There was no significant difference between sham WT and Cav‐3 OE mice for diastolic or systolic left ventricular volume (Figure 1b,c), stroke volume or ejection fraction (Figure 1d,e), cardiac output (WT: 19.3 ± 4.2 ml min^−1^, *N* = 7; Cav‐3 OE: 19.5 ± 2.8 ml min^−1^, *N* = 6; *P *= 0.92; not shown) or left ventricular mass (WT: 150.3 ± 24.8 mg, *N* = 7; Cav‐3 OE: 141.3 ± 21.2 mg, *N* = 6; *P *= 0.50; not shown). Following TAC, diastolic and systolic left ventricular volume increased, and ejection fraction decreased, in both WT and Cav‐3 OE mice (Figure 1b,c,e), while stroke volume (Figure 1d) and thus cardiac output (WT: 16.3 ± 6.1 ml min^−1^, *N* = 7, *P *= 0.30; Cav‐3 OE: 11.5 ± 3.3 ml min^−1^, *N* = 6, *P *< 0.01) decreased significantly in Cav‐3 OE, but not WT mice. However, none of the measurements of cardiac function were significantly different between WT and Cav‐3 OE mice following TAC. Thus, it appears that Cav‐3 OE has little effect on *in vivo* cardiac function either in sham animals or following TAC. However, although TAC increased left ventricular mass in both types of mouse (to 339.6 ± 33.9 mg in WT, *N* = 7, *P *< 0.001; and to 271.6 ± 24.7 mg in Cav‐3 OE, *N* = 6, *P *< 0.001), left ventricular mass was significantly smaller (*P *< 0.01) in Cav‐3 OE than in WT mice following TAC. Heart rate was not significantly different between groups (WT: sham 446.9 ± 7.3 bpm, *N* = 7; TAC 444.7 ± 20.0 bpm, *N* = 7, *P *= 0.92; Cav‐3 OE: sham 447 ± 27 bpm, *N* = 6; TAC 453 ± 54 bpm, *N* = 6, *P *= 0.81).

Consistent with the changes observed *in vivo*, following TAC both WT and Cav‐3 OE mice showed a significant increase in wet heart weight (WT: sham 210 ± 34 mg, *N* = 7, TAC 401 ± 77 mg, *N* = 8, *P *< 0.001; Cav‐3 OE: sham 227 ± 50 mg, *N* = 6, TAC 362 ± 35 mg, *N* = 6, *P *< 0.001) and wet lung weight (WT: sham 146 ± 22 mg, *N* = 7, TAC 345 ± 127 mg, *N* = 8, *P *< 0.01; Cav‐3 OE: sham 173 ± 19 mg, *N* = 6, TAC 427 ± 134 mg, *N* = 6, *P *< 0.001), but no change in body weight (WT: sham 28.5 ± 1.5 g, *N* = 7, TAC 28.3 ± 2.6 g, *N* = 8, *P *= 0.86; Cav‐3 OE: sham 27.4 ± 1.9 g, *N* = 6; TAC 26.6 ± 4.6 g, *N* = 6, *P *= 0.68) or tibia length (WT: sham 20.4 ± 0.6 mm, *N* = 7; TAC 19.8 ± 0.7 mm, *N* = 8, *P *= 0.10; Cav‐3 OE: sham 19.8 ± 0.9 mm, *N* = 6; TAC 19.6 ± 0.9 mm, *N* = 6, *P *= 0.62). Thus, in both WT and Cav‐3 OE mice, TAC resulted in cardiac hypertrophy, indicated by a significant increase in heart weight:tibia length ratio (HW:TL; Figure 1f), and pulmonary congestion, a symptom of congestive heart failure, indicated by a significant increase in lung weight:tibia length ratio (LW:TL; Figure 1g); however, HW:TL following TAC was significantly smaller in Cav‐3 OE than in WT mice (Figure 1f). These data suggest, therefore, that Cav‐3 OE *per se* has little effect on cardiac structure or function, but reduces the hypertrophy observed following TAC. The magnitude of these changes was similar to that reported previously in Cav‐3 OE mice following TAC (Horikawa et al., 2011, and see below).

### 3.2 The effect of TAC on cell morphology

**Figure 2:**
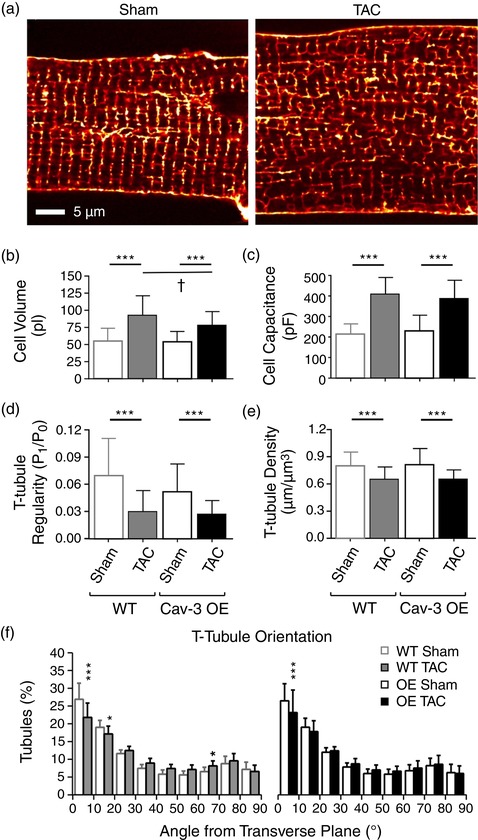
Morphology of isolated myocytes. (a) Confocal images of t‐tubules and surface sarcolemma stained with di‐8‐ANEPPS from representative sham (left) and TAC (right) Cav‐3 OE myocytes. Scale bar: 5 μm. (b,c) Cell volume (b) and cell capacitance (c) from sham (*n*/*N* = 19/5) and TAC (*n*/*N* = 22/5) Cav‐3 OE myocytes (right) compared with WT sham (*n*/*N* = 41/10) and TAC (*n*/*N* = 21/5) myocytes (left). (d–f) Analysis of t‐tubule organization from di‐8‐ANEPPS‐labelled sham (*n*/*N* = 23/5) and TAC (*n*/*N* = 27/5) Cav‐3 OE myocytes (right) and WT sham (*n*/*N* = 40/8) and TAC (*n*/*N* = 21/5) myocytes (left). (d) T‐tubule power (*P*_1_/*P*_0_). (e,f) T‐tubule density (μm μm^−3^; e) and T‐tubule orientation (degrees from transverse plane; f) in WT and Cav‐3 OE myocytes. ***P < 0.001 between treatments for a given phenotype (WT or Cav‐3 OE); †*P *< 0.05 between phenotypes for a given treatment (sham or TAC). The WT data have been published previously (Bryant et al., 2018a)

Figure 2a shows exemplar confocal images of isolated Cav‐3 OE myocytes stained with di‐8‐ANEPPS, showing an increase in cell width, suggesting cellular hypertrophy, and disruption of t‐tubule structure following TAC. Mean data show that in both WT and Cav‐3 OE myocytes, TAC increased cell length (WT: sham 166.1 ± 16.7 μm, *n*/*N* = 41/10, TAC 192.0 ± 57.9 μm, *n*/*N* = 21/5, *P *< 0.01; Cav‐3 OE: sham 149.3 ± 16.1 μm, *n*/*N* = 19/5, TAC 178.3 ± 15.6 μm, *n*/*N* = 22/5, *P *< 0.001) and width (WT: sham 35.2 ± 5.3 μm, *n*/*N* = 41/10, TAC 43.1 ± 3.9 μm, *n*/*N* = 21/5, *P *< 0.001; Cav‐3 OE: sham 36.7 ± 4.5 μm, *n*/*N* = 19/5, TAC 40.5 ± 5.0 μm, *n*/*N* = 22/5, *P *< 0.05), and thus calculated cell volume (Figure 2b). Although Cav‐3 OE had no effect on cell volume in the absence of TAC, it was significantly smaller in Cav‐3 OE than in WT myocytes following TAC (Figure 2b). Cell capacitance (a function of membrane area) also increased significantly in both WT and Cav‐3 OE myocytes (Figure 2c), consistent with the development of cellular hypertrophy in response to pressure overload.

Analysis of t‐tubule structure using 2D FFT showed a significant decrease in t‐tubule regularity in both WT and Cav‐3 OE myocytes following TAC (*P*_1_/*P*_0_, Figure 2d). Since *P*_1_/*P*_0_ depends, *inter alia*, on t‐tubule density and orientation, further detailed analysis in 3D was performed, which showed that this reduction in regularity was due, at least in part, to a significant decrease in t‐tubule density (Figure 2e) and changes in tubule orientation, with a decrease in the fraction of transverse (0–15°) tubules (Figure 2f). However, the changes in t‐tubule structure were not significantly different between WT and Cav‐3 OE myocytes.

**Figure 3:**
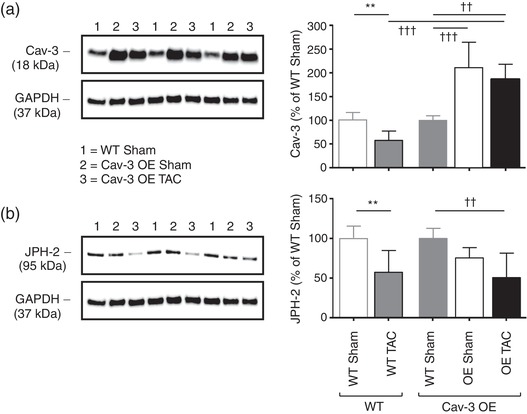
Effect of Cav‐3 OE and TAC on Cav‐3 and JPH‐2 protein expression. (a) Left panel, exemplar western blots of Cav‐3 (18 kDa) and GAPDH (37 kDa) in isolated myocyte lysates from three WT sham (lanes 1), three Cav‐3 OE sham (lanes 2) and three Cav‐3 OE TAC (lanes 3) mice; Right panel, densitometry analysis of Cav‐3 western blots (*N* = 5 animals in each group in duplicate), compared with previously published data (Bryant et al., 2018a) showing the effect of TAC on Cav‐3 expression in WT mice (left two bars). (b) Left panel, exemplar western blots of JPH‐2 (95 kDa) and GAPDH (37 kDa); right panel, densitometry analysis of JPH‐2 western blots (*N* = 5 animals in each group in duplicate), compared with previously published data (Bryant et al., 2018a) showing the effect of TAC on JPH‐2 expression in WT mice (left two bars). The blots in the left panels are from the same gels which were stripped and re‐probed for the different proteins and were therefore obtained sequentially (see Methods). Data in each group (WT or Cav‐3 OE) in the right panels are expressed as a percentage of the mean of the WT data in that group. **P < 0.01, between treatments for a given phenotype (WT or Cav‐3 OE); ††*P *< 0.01, †††P < 0.001 between phenotypes

To ensure that Cav‐3 expression was increased in OE mice, we used western blotting. Figure 3a shows exemplar blots (left) and corresponding mean densitometric analysis (right) showing that Cav‐3 expression was increased ∼2‐fold in OE compared to WT myocytes. Cav‐3 expression was not significantly altered following TAC in Cav‐3 OE myocytes, so that its expression level remained higher than in sham and TAC WT myocytes. Since t‐tubule structure was altered following TAC in Cav‐3 OE myocytes, we also investigated the expression of JPH‐2, which has been implicated in t‐tubule and dyad formation. Figure 3b shows exemplar blots (left) and corresponding mean densitometric analysis (right) showing that JPH‐2 was not significantly altered by Cav‐3 OE but decreased following TAC in both WT and Cav‐3 OE myocytes.

In summary, Cav‐3 OE appears to have little effect on cardiac and cell morphology, and *in vivo* cardiac function, and the response to TAC was qualitatively similar in WT and Cav‐3 OE mice; however, both heart weight and cell volume were significantly smaller in Cav‐3 OE mice than in WT, following TAC.

### 3.3 *I*_Ca_ distribution and regulation

**Figure 4:**
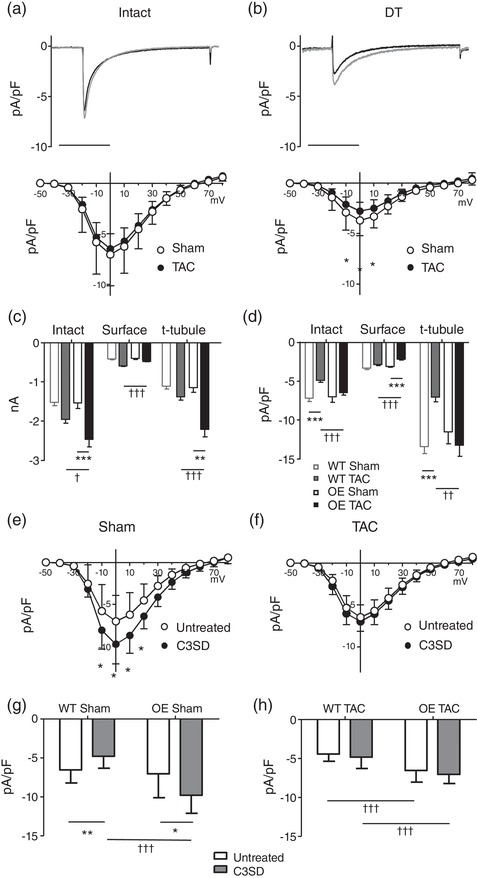
Surface and t‐tubular *I*_Ca_. (a) Top panel, exemplar records of *I*_Ca_ from intact sham (black) and TAC (grey) Cav‐3 OE myocytes. Bottom panel, corresponding mean *I*_Ca_ density–voltage relationships from sham (*n*/*N* = 19/5) and TAC (*n*/*N* = 22/5) Cav‐3 OE myocytes. Two‐way repeated measures ANOVA: mV *P *< 0.001, TAC *P *= 0.4, interaction *P *< 0.9. (b) Top panel, exemplar records of *I*_Ca_ from detubulated (DT) sham (black) and TAC (grey) Cav‐3 OE myocytes. Bottom panel: corresponding mean *I*_Ca_ density–voltage relationships from DT sham (*n*/*N* = 19/5) and TAC (*n*/*N* = 21/5) Cav‐3 OE myocytes. Two‐way repeated measures ANOVA: mV *P *< 0.001, TAC *P *< 0.001, interaction *P *< 0.001. (c,d) Absolute *I*_Ca_ (c) and *I*_Ca_ density (d) at 0 mV in intact Cav‐3 OE myocytes, and at the cell surface and t‐tubules (obtained as described in Methods), compared with previously published data (Bryant et al., 2018a) from WT mice. **P < 0.01, ***P < 0.001 between treatments for a given phenotype (WT or Cav‐3 OE); †*P *< 0.05, ††*P *< 0.01, †††*P* < 0.001 between phenotypes for a given treatment (sham or TAC). (e) Mean *I*_Ca_ density–voltage relationships from sham untreated (*n*/*N* = 19/5) and C3SD treated (*n*/*N* = 12/3) Cav‐3 OE myocytes. Two‐way repeated measures ANOVA: mV *P *< 0.001, C3SD *P *= 0.014, interaction *P *< 0.001). (f) Mean *I*_Ca_ density–voltage relationships from TAC untreated (*n*/*N* = 22/5) and C3SD treated (*n*/*N* = 17/5) Cav‐3 OE myocytes. Two‐way repeated measures ANOVA: mV *P *< 0.001, C3SD *P *= 0.13, interaction *P *= 0.4. (g) Mean *I*_Ca_ density at 0 mV in sham untreated and C3SD treated Cav‐3 OE myocytes, compared with previously published data (Bryant et al., 2018a; see Methods) from sham untreated (*n*/*N* = 16/5) and C3SD treated (*n*/*N* = 17/5) WT myocytes. Two‐way ANOVA: OE *P *< 0.001, C3SD *P *= 0.4, interaction *P *< 0.001. (h) Mean *I*_Ca_ density at 0 mV in TAC untreated and C3SD treated Cav‐3 OE myocytes, compared with previously published data (Bryant et al., 2018a; see Methods) from TAC untreated (*n*/*N* = 19/5) and C3SD treated (*n*/*N* = 15/5) WT myocytes. Two‐way ANOVA: OE *P *< 0.001, C3SD *P *= 0.14, interaction *P *= 0.84. *P < 0.05, **P < 0.01 between treatments for a given phenotype (WT or Cav‐3 OE); †††P < 0.001 between phenotypes for a given treatment

To determine the distribution of *I*_Ca_ between the surface and t‐tubular membranes, *I*_Ca_ was measured in intact and DT myocytes. Figure 4 shows exemplar records of *I*_Ca_ recorded at 0 mV from intact (Figure 4a, top) and DT (Figure 4b, top) myocytes isolated from sham and TAC Cav‐3 OE hearts, with the corresponding mean current density–voltage relationships shown below. Absolute *I*_Ca_ and *I*_Ca_ density were not significantly different in WT and Cav‐3 OE myocytes (Figure 4c,d). However, the increase in cell capacitance caused by TAC in WT mice (Figure 2c) occurred with little change in absolute *I*_Ca_ (Figure 4c), so that *I*_Ca_ density decreased (Figure 4d); in contrast, in Cav‐3 OE myocytes, the TAC‐induced increase in cell capacitance (Figure 2c) was accompanied by an increase in absolute *I*_Ca_ amplitude (Figure 4c) so that *I*_Ca_ density was maintained following TAC (Figure 4d).

Detubulation decreased *I*_Ca_ density in both sham (*P *< 0.001) and TAC (*P *< 0.001) Cav‐3 OE myocytes (cf. Figs 4a,b). However, there was no significant difference in absolute *I*_Ca_ amplitude at the surface membrane of sham and TAC Cav‐3 OE myocytes (Figure 4C), so that the increase in cell size (and thus capacitance) following TAC resulted in a significant decrease in *I*_Ca_ density at the cell surface (Figure 4d); this contrasts with the small increase in absolute *I*_Ca_ at the surface membrane of WT myocytes following TAC, so that *I*_Ca_ density at the surface membrane is unaltered following TAC in these cells (Figure 4c,d). The lack of change of *I*_Ca_ density in intact Cav‐3 OE myocytes following TAC, despite a decrease at the surface membrane, suggests that *I*_Ca_ density at the t‐tubule membrane is increased. Calculation of t‐tubular *I*_Ca_ showed that absolute *I*_Ca_ is significantly increased so that *I*_Ca_ density is maintained in the t‐tubules of Cav‐3 OE myocytes following TAC (Figure 4c,d), in contrast to the lack of change of absolute *I*_Ca_ and thus decrease in t‐tubular *I*_Ca_ density observed in WT myocytes following TAC (Figure 4c,d). Thus, *I*_Ca_ density at the cell surface decreases and t‐tubular *I*_Ca_ density is preserved in Cav‐3 OE mice following TAC, whereas WT mice show no change at the cell surface and a decrease in t‐tubular *I*_Ca_ density in response to TAC.

We have previously shown that incubating cells with C3SD, which mimics the scaffolding domain of Cav‐3 and has no effect on cell capacitance (Bryant et al., 2014), decreases *I*_Ca_ density in intact WT control myocytes (Bryant et al., 2018a; Kong et al., 2017), and that this effect is lost following Cav‐3 KO and in TAC‐induced HF (Bryant et al., 2018a). To investigate whether changes in this regulatory pathway might underlie the different *I*_Ca_ distributions observed following TAC in OE mice, we determined the response to C3SD in myocytes from sham and TAC Cav‐3 OE mice. Figure 4e,g show that in sham Cav‐3 OE myocytes *I*_Ca_ density was *increased* by pre‐treatment with C3SD, as reported previously in myocytes from unoperated Cav‐3 OE mice (Kong et al., 2017) but in contrast to the decrease of *I*_Ca_ density observed in sham WT myocytes in response to C3SD (Bryant et al., 2018a; Figure 4g). However, C3SD had no effect on *I*_Ca_ density in Cav‐3 OE myocytes following TAC (Figure 4f,h); this loss of response to C3SD following TAC is similar to that reported in WT myocytes following TAC (Bryant et al., 2018a; Figure 4h). This suggests that this regulatory pathway is lost in both cell types and is not, therefore, responsible for the different distribution of *I*_Ca_ density observed in OE and WT myocytes following TAC.

### 3.4 Ca^2+^ release following TAC

**Figure 5:**
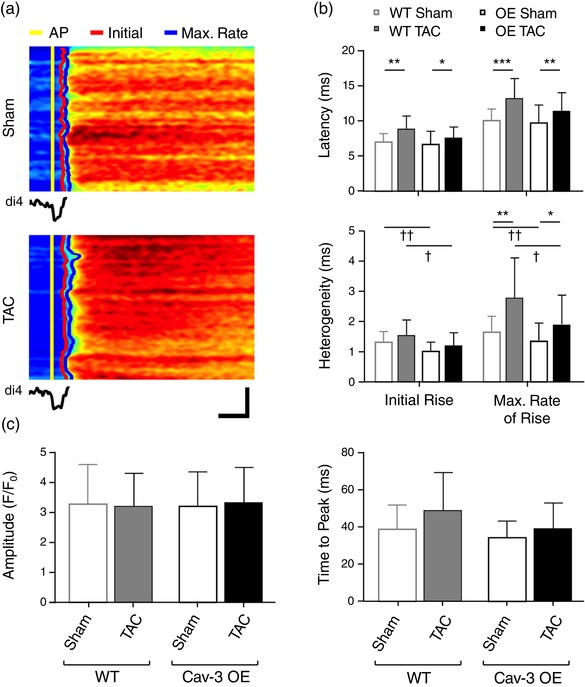
Local Ca^2+^ release and systolic Ca^2+^ transient. (a) Line‐scan images of the rising phase of the Ca^2+^ transient and (below) the associated average t‐tubular di‐4‐AN(F)EPPTEA signal in representative sham (top) and TAC (bottom) Cav‐3 OE myocytes. Horizontal scale bar: 10 ms; vertical scale bar: 5 μm. The time of the AP upstroke, initial rise of Ca^2+^ and maximum rate of rise of Ca^2+^ have been marked in yellow, red and blue, respectively. (b) Mean latency and heterogeneity of SR Ca^2+^ release in sham (*n*/*N* = 28/5) and TAC (*n*/*N* = 18/4) Cav‐3 OE myocytes compared with WT sham (*n*/*N* = 43/12) and TAC (*n*/*N* = 12/3) myocytes. (c) Whole cell Ca^2+^ transient amplitude (*F*/*F*_0_) and time to peak (ms) measured from Ca^2+^ transients of sham (*n*/*N* = 25/5) and TAC (*n*/*N* = 22/4) Cav‐3 OE myocytes compared with WT sham (*n*/*N* = 53/13) and TAC (*n*/*N* = 14/3) myocytes. *P < 0.05, **P < 0.01, ***P < 0.001 between treatments for a given phenotype (WT or Cav‐3 OE); †*P *< 0.05, ††*P *< 0.01 between phenotypes for a given treatment (sham or TAC). The WT data have been published previously (Bryant et al., 2018a)

To determine whether the preservation of t‐tubular *I*_Ca_ in Cav‐3 OE myocytes helps to maintain Ca^2+^ release, we investigated the latency and heterogeneity of Ca^2+^ release along a single t‐tubule from the time of membrane depolarization (Figure 5a), which showed that Cav‐3 OE had no significant effect on latency, nor did it affect the increase in latency of both the initial and maximum rate of rise of Ca^2+^ observed following TAC (Figure 5b, top), suggesting that TAC‐induced impairment of local Ca^2+^ release is unaffected by Cav‐3 OE. However, Cav‐3 OE caused a significant decrease in the heterogeneity of Ca^2+^ release compared to WT myocytes, and although heterogeneity increased following TAC in both cell types, it remained smaller in Cav‐3 OE myocytes (Figure 5b, bottom) suggesting more uniform Ca^2+^ release along the t‐tubules following Cav‐3 OE. However, TAC had little effect on the early (release) phase of the whole cell Ca^2+^ transient, which was not significantly different in WT and Cav‐3 OE myocytes and showed no change in either time to peak or amplitude (Figure 5c).

### 3.5 Comparison with HF in WT myocytes

**Figure 6:**
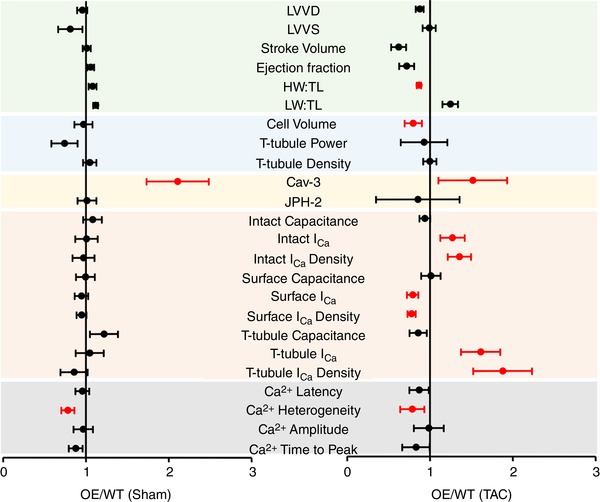
Comparison of WT and OE data. Plots showing the ratio of data from Cav‐3 OE mice to those from WT mice, ± 95% confidence intervals, in sham (left) and in HF following TAC (right). The WT data have been published previously (Bryant et al., 2018a). The *x*‐axis shows the change relative to WT: an increase in Cav‐3 OE mice compared to WT results in a value >1, while a decrease results in a value <1. The coloured bands delineate different groups of data that correspond to those shown in Figs 1 (green, top) to 5 (grey, bottom). The data in red are significantly different in Cav‐3 OE and WT mice (statistical analysis performed using original data). LVVD, left ventricular volume at diastole, μl; LVVS, left ventricular volume at systole, μl; stroke volume, μl; ejection fraction, %; HW:TL, heart weight to tibial length ratio, mg mm^−1^; LW:TL, lung weight to tibial length ratio, mg mm^−1^; cell volume, pl; t‐tubule power, *P*_1_/*P*_0_; t‐tubule density, μm μm^−3^; Cav‐3, caveolin‐3 expression, %; JPH‐2, junctophilin expression, %; intact capacitance, whole cell capacitance, pF; intact *I*_Ca_, whole‐cell *I*_Ca_ amplitude at 0 mV, pA; intact *I*_Ca_ density, whole‐cell *I*_Ca_ density at 0 mV, pA pF^−1^; surface capacitance, surface membrane capacitance, pF; surface *I*_Ca_, surface membrane *I*_Ca_ amplitude at 0 mV, pA; surface *I*_Ca_ density, surface membrane *I*_Ca_ density at 0 mV, pA pF^−1^; t‐tubule capacitance, t tubule membrane capacitance, pF; t‐tubule *I*_Ca_, t‐tubule membrane *I*_Ca_ amplitude at 0 mV, pA; t‐tubule *I*_Ca_ density, t‐tubule membrane *I*_Ca_ density, pA pF^−1^; Ca^2+^ latency, calcium transient latency from action potential depolarization, ms; Ca^2+^ heterogeneity, calcium transient heterogeneity during upstroke, ms; Ca^2+^ amplitude, calcium transient amplitude, *F*/*F*_0_; Ca^2+^ time to peak, ms

Figure 6 shows the ratio of the data from Cav‐3 OE mice to those from WT mice, in sham (left) and in mice in HF following TAC (right). These data show that Cav‐3 OE had little effect on measurements from sham hearts and myocytes, consistent with previous work showing little effect of Cav‐3 OE on cardiac morphology or function in the absence of TAC (Horikawa et al., 2011; Kong et al., 2017; Markandeya et al., 2015). However, following TAC, HW:TL and cell volume were significantly smaller in OE than in WT, consistent with previous work (Horikawa et al., 2011; Markandeya et al., 2015). In addition, *I*_Ca_ amplitude and density were greater in intact OE than in WT myocytes, as a result of a small decrease at the cell surface but a large increase at the t‐tubules. Thus, the decrease in t‐tubular *I*_Ca_ normally observed following TAC (Bryant et al., 2018a) is prevented by Cav‐3 overexpression, and although Cav‐3 OE exerts an anti‐hypertrophic effect and increases *I*_Ca_, these effects only become apparent following TAC.

## 4 DISCUSSION

The present data show that TAC caused qualitatively similar changes in Cav‐3 OE mice to those reported previously in WT mice (Bryant et al., 2018a): cardiac hypertrophy and failure, with disrupted cell structure and function. However, the cardiac and cellular hypertrophy associated with TAC were smaller in OE animals and t‐tubular *I*_Ca_ density was maintained, although *I*_Ca_ density at the surface membrane decreased; this contrasts with the decrease in t‐tubular *I*_Ca_ density with no change at the cell surface observed in WT myocytes. Thus Cav‐3 OE appears to confer limited but specific protection against the effects of TAC.

### 4.1 Cardiac structure and function

Echocardiography showed that Cav‐3 OE had little effect on cardiac structure or function in sham animals (Figure 1). Following TAC, diastolic and systolic left ventricular volume and left ventricular mass increased, and *post mortem* measurements also showed an increase in HW:TL and LW:TL in Cav‐3 OE mice. However, although cardiac function was not significantly different in WT and Cav‐3 OE mice following TAC, the hypertrophic response to TAC was smaller in Cav‐3 OE than in WT mice. These changes are similar to those reported previously following 4 weeks TAC in Cav‐3 OE mice (Horikawa et al., 2011) and suggest that Cav‐3 OE has maintained anti‐hypertrophic effects following TAC, consistent with the idea that Cav‐3 inhibits the hypertrophic p42/44 mitogen‐activated protein kinase (Woodman et al., 2002) and calmodulin‐dependent calcineurin/nuclear factor of activated T cells (Markandeya et al., 2015) pathways.

Following TAC, cardiac function was not significantly different, and there were similar decreases in ejection fraction, in WT and Cav‐3 OE mice. However, stroke volume and cardiac output decreased significantly in Cav‐3 OE but not in WT mice following TAC. This implies that an increase in heart size in WT (cf. the larger scatter in Figure 1b) compared to Cav‐3 OE mice helped to maintain stroke volume and thus cardiac output despite similar decreases in ejection fraction. Thus, the anti‐hypertrophic effect of Cav‐3 OE may impair the ability of the heart to maintain cardiac output.

Previous work has shown that 4 weeks’ TAC caused only a small (not significant) decrease in cardiac function in Cav‐3 OE mice, but a significant decrease in WT mice (Horikawa et al., 2011; Markandeya et al., 2015). The contrast with the current work may be due to the longer (8 weeks) exposure to TAC in the present study. Taken together these data suggest that, while the anti‐hypertrophic effect of Cav‐3 is maintained, the deleterious effect on cardiac function, while slowed in onset, can still occur in Cav‐3 OE mice following TAC. However, the observation that Cav‐3 OE has little effect on either the size or the function of the heart in the absence of TAC (Figure 6; Horikawa et al., 2011; Markandeya et al., 2015) suggests that Cav‐3 expression is normally sufficient to inhibit hypertrophy and enable normal ECC.

### 4.2 Cell structure

In the absence of TAC, Cav‐3 OE had little effect on cell size or structure, as reported previously (Kong et al., 2017), but cells were larger and t‐tubule structure was disrupted following TAC, as in WT myocytes (Bryant et al., 2018a). However, the increase in cell volume following TAC was significantly smaller in OE than in WT myocytes, providing a mechanism for the reduced hypertrophy observed in the whole heart and consistent with the suggestion that caveolin inhibits hypertrophic pathways (Galbiati et al., 1998; Markandeya et al., 2015; Woodman et al., 2002). Knockout and loss‐of‐function mutations of Cav‐3 are associated with hypertrophic cardiomyopathy, further supporting a role for Cav‐3 as an inhibitor of cardiac hypertrophic signalling pathways (Hayashi et al., 2004; Woodman et al., 2002).

### 4.3 Cell function

Cav‐3 OE had little effect on the function of myocytes from sham hearts. However, the distribution of *I*_Ca_ was markedly different in OE and WT myocytes following TAC. TAC‐induced heart failure in WT mice is associated with a decrease in t‐tubular *I*_Ca_ density due to cellular hypertrophy with no change in absolute current, and no change in *I*_Ca_ density at the cell surface (Bryant et al., 2018a). In contrast, in OE myocytes, there was no change in t‐tubular *I*_Ca_ density following TAC, because absolute current increased proportionally with membrane area, but *I*_Ca_ density at the cell surface decreased, since absolute *I*_Ca_ was unchanged despite cellular hypertrophy. Thus, following TAC, Cav‐3 OE maintains *I*_Ca_ density at the t‐tubular membrane but not at the cell surface.

Previous work has shown that chronically decreasing Cav‐3 expression, via either KO or TAC, leads to decreased t‐tubular *I*_Ca_ density as a result of an increase in membrane area (Bryant et al., 2018a), whereas acute disruption of Cav‐3 activity using C3SD peptide decreases *I*_Ca_ with no change of capacitance (Bryant et al., 2018a; Kong et al., 2017). However, in agreement with previous work (Kong et al., 2017), the present data show that C3SD *increases I*_Ca_ in OE myocytes although, as in WT myocytes, this regulation was lost following TAC.

These data suggest that Cav‐3 alters *I*_Ca_ density by (at least) two mechanisms. The first is by altering cell growth. It has previously been suggested that Cav‐3 is anti‐hypertrophic (Woodman et al., 2002), consistent with the observations *in vivo*, and in KO and WT TAC myocytes. Previous work has shown that hypertrophic pathways can be activated by Ca^2+^ entry via LTCCs (Gao et al., 2012), although LTCCs localized to caveolae do not appear to contribute to hypertrophic signalling in mouse ventricular myocytes (Correll et al., 2017); thus Cav‐3 may inhibit hypertrophic signalling by altering the distribution of LTCCs, and thus *I*_Ca_. The present study suggests that Cav‐3 OE does not augment the anti‐hypertrophic effect of basal Cav‐3 levels in sham myocytes, since cell volume and capacitance in the absence of TAC are similar in WT and OE myocytes. However, Cav‐3 OE does appear to be anti‐hypertrophic following TAC, when OE myocytes are smaller than those from WT. Thus, it appears that Cav‐3 levels are normally sufficient to inhibit hypertrophy, and only become insufficient to do so following TAC, either because Cav‐3 levels decrease and/or because of stimulation of hypertrophic pathways. This anti‐hypertrophic effect of Cav‐3 following TAC will help to maintain *I*_Ca_ density. However, the observation that absolute t‐tubular *I*_Ca_ increases suggests additional effects of Cav‐3 OE, in particular modulation of acute signalling pathways. Previous work suggests that Cav‐3 helps localize *I*_Ca_ to the t‐tubules, and C3SD decreases t‐tubular *I*_Ca_ in WT myocytes, although this effect is lost following TAC (Bryant et al., 2018a). It has been suggested that Cav‐3‐dependent stimulation of *I*_Ca_ is due to co‐localization of LTCCs with components of the protein kinase A pathway (Bryant et al., 2014). However, recent work has shown LTCC clustering leading to co‐operative gating (Ghosh et al., 2018); if Cav‐3 plays a role in this process, loss of Cav‐3 activity would be expected to decrease *I*_Ca_. The present study shows that C3SD *increases I*_Ca_ in sham Cav‐3 OE myocytes, suggesting that the levels of Cav‐3 expression achieved in OE myocytes may *inhibit* these Cav‐3 dependent pathways. Such inhibition could occur as the result of autoinhibition or because there is abnormally located Cav‐3 in OE myocytes which competes with normally localized proteins. Reducing Cav‐3 expression, activity or regulation, via TAC or C3SD, may relieve this inhibitory effect to produce the increases in *I*_Ca_ observed in these conditions; the loss of effect of C3SD on *I*_Ca_ following TAC is also indicative of such loss of Cav‐3 dependent regulation.

In contrast to t‐tubular *I*_Ca_, *I*_Ca_ density at the cell surface decreased following TAC in Cav‐3 OE mice, due to cellular hypertrophy with little change in absolute *I*_Ca_. Thus, following TAC, in OE myocytes t‐tubular *I*_Ca_ appears to be maintained at the expense of *I*_Ca_ at the surface membrane, whereas in WT myocytes t‐tubular *I*_Ca_ density decreases with no change at the cell surface. This suggests that changes in Cav‐3 expression occur predominantly at the t‐tubules, where it competes with that at the surface membrane to bind the proteins which localize *I*_Ca_.

Regardless of the mechanism underlying the maintenance of t‐tubular *I*_Ca_, the latency and heterogeneity of local Ca^2+^ release at the t‐tubule still increased following TAC which, with the disruption of t‐tubule morphology also observed following TAC, would be expected to desynchronize Ca^2+^ release and thus impair contraction. However, the whole cell Ca^2+^ transient showed little change, suggesting that it is dominated by other factors and is not responsible for the impaired cardiac performance observed following TAC. However, the Ca^2+^ transient was monitored at a low stimulation frequency (0.2 Hz) compared with the mouse's normal heart rate, to enable comparison with *I*_Ca_, which was recorded at this frequency to allow recovery from inactivation between voltage clamp pulses. It remains possible, therefore, that changes in local Ca^2+^ release may decrease Ca^2+^ transient amplitude, and thus impair cardiac performance, at physiological frequencies. However, the impairment occurred in the presence of increased heart size, so that reduced stroke volume and ejection fraction do not necessarily imply reduced contractility because myocyte contraction will have to overcome the higher wall tension that will result from the increase in heart size (law of Laplace).

The lack of effect of Cav‐3 OE on the latency of Ca^2+^ release despite the recovery of t‐tubular *I*_Ca_ suggests that the decrease in *I*_Ca_ is not the primary cause of disruption of Ca^2+^ release following TAC. This may be because of redundancy in the Ca^2+^‐induced Ca^2+^ release process (Cannell, Berlin, & Lederer, 1987) in mice, and/or because the LTCCs, and thus *I*_Ca_, which are regulated by Cav‐3 are predominantly extra‐dyadic (Glukhov et al., 2015; Sanchez‐Alonso et al., 2016), which could also explain why similar decreases in *I*_Ca_ amplitude produced by Ca^2+^ channel blockers, which will affect all Ca^2+^ channels, inhibit release (Bryant et al., 2015). The TAC‐induced disruption of Ca^2+^ release may, therefore, be due predominantly to disruption of the dyad (Louch et al., 2013), consistent with the observed decrease in JPH‐2 and with recent work showing dispersion of RyR clusters in rat myocytes in HF (Kolstad et al., 2018), so that RyR dispersion and loss of t‐tubular *I*_Ca_ may have summative effects that impair Ca^2+^ release along t‐tubules. Interestingly, however, Cav‐3 OE decreased the heterogeneity of Ca^2+^ release in both sham and TAC myocytes, suggesting that Cav‐3 may increase the uniformity of t‐tubular Ca^2+^ release by altering the distribution of *I*_Ca_ or the response to *I*_Ca_ along the t‐tubule.

## 5 SUMMARY

These data show that Cav‐3 OE alone has little effect on the structure or function of either the whole heart or ventricular myocytes. However, following TAC, Cav‐3 OE is anti‐hypertrophic and helps to maintain t‐tubular *I*_Ca_; this is not secondary to the smaller increase in t‐tubular membrane area in OE myocytes, because absolute *I*_Ca_ increases. This attenuation of the TAC phenotype by Cav‐3 OE is consistent with previous work suggesting that Cav‐3 plays a role in the local regulation of *I*_Ca_ and is anti‐hypertrophic, but it remains unclear whether these effects are direct or secondary.

## AUTHOR CONTRIBUTIONS

All laboratory experiments were performed at the University of Bristol. S.M.B., C.H.T.K., D.M.R., H.H.P., A.F.J., M.B.C. and C.H.O. conceived the study and designed the project methods. S.M.B., C.H.T.K. and J.J.W. contributed to acquisition, analysis or interpretation of data and S.M.B., C.H.T.K., J.J.W., D.M.R., H.H.P., A.F.J., M.B.C. and C.H.O. contributed to the drafting of the manuscript and its revision. All authors approved the final version of the manuscript and agree to be accountable for all aspects of the work in ensuring that questions related to the accuracy or integrity of any part of the work are appropriately investigated and resolved. All persons designated as authors qualify for authorship, and all those who qualify for authorship are listed.
